# Analysis of Impact of Natural Ventilation Strategies in Ventilation Rates and Indoor Environmental Acoustics Using Sensor Measurement Data in Educational Buildings

**DOI:** 10.3390/s21186122

**Published:** 2021-09-12

**Authors:** María L. de la Hoz-Torres, Antonio J. Aguilar, Diego P. Ruiz, María Dolores Martínez-Aires

**Affiliations:** 1 Department of Applied Physics, University of Granada, Av. Severo Ochoa s/n, 18071 Granada, Spain; mlhoz@ugr.es (M.L.d.l.H.-T.); antojes@ugr.es (A.J.A.); druiz@ugr.es (D.P.R.); 2 Department of Building Construction, University of Granada, Av. Severo Ochoa s/n, 18071 Granada, Spain

**Keywords:** buildings, ventilation rate, indoor air quality, natural ventilation, COVID-19

## Abstract

Indoor environmental conditions can significantly affect occupants’ health and comfort. These conditions are especially important in educational buildings, where students, teachers and staff spend long periods of the day and are vulnerable to these factors. Recently, indoor air quality has been a focus of attention to ensure that disease transmission in these spaces is minimised. In order to increase the knowledge in this field, experimental tests have been carried out to characterise the impact of natural ventilation strategies on indoor air quality and the acoustic environment. This study has evaluated three ventilation scenarios in four different classrooms in buildings of the University of Granada, considering different window and door opening configurations. Ventilation rates were estimated using the CO_2_ Decay Method, and background noise recordings were made in each classroom for acoustic tests. Results show that specific natural ventilation strategies have a relevant impact that is worth considering on the background noise in indoor spaces. In this sense ventilation rates provided by the different configurations varied between 3.7 and 39.8 air changes per hour (ACH) and the acoustic tests show a background noise ranging from 43 to 54 dBA in these scenarios. Consequently, managers and teachers should take into account not only the ACH, but also other collateral impacts on the indoor environmental conditions such as the thermal comfort or the acoustic environment.

## 1. Introduction

Since people spend more than 80% of their time in indoor environments [1], if the indoor conditions are deficient, the health and comfort of the occupants may be affected [2]. Building design and its characteristics are important factors of indoor conditions and, hence, the satisfaction levels of the occupants [3]. Indoor environmental quality (IEQ) is defined as an indication that relates the health and well-being of the occupants of interior spaces with the quality of the building’s environment [4].

The IEQ is essential in educational buildings, which are typically designed for high occupancy for long periods of the day [5,6]. In particular, a good indoor air quality (IAQ) is crucial to provide a healthy, safe, productive and comfortable environment [7]. Students, teachers and other school staff are vulnerable to the impact of poor IAQ in these spaces, where concentration and intellectual work is required. Indoor air pollutants (i.e., inorganic/organic gases and biological and non-biological particles) accumulate more easily in indoor environments as a result of the building envelopes which intentionally separate occupants from the outside [8]. Exposure to air pollutants may cause a risk of short- and long-term health problems, such as several respiratory diseases [9,10], cardiovascular disease [11], irritated eyes or nose, blocked nose, headaches and so forth [12]. In addition, poor IAQ may affect the comfort, productivity and academic achievement of students [6,13,14]. Therefore, IAQ is of particular concern in teaching‒learning spaces.

These circumstances determine that one of the most demanding challenges facing educational building administrators is IAQ managing [15]. An adequate ventilation rate (VR) is one of the key elements to avoid compromising the IAQ since providing outdoor air ventilation dilutes internally generated contaminants to levels that do not cause health and comfort problems [16]. The analysis of the VR based on measured studies and the adequately characterised ventilation design of buildings are critical for assessing and interpreting IAQ [17,18]. Selecting an appropriate ventilation strategy is essential for meeting the requirements for good IAQ. International guidelines, standards and building codes state a minimum VR in buildings [19,20,21,22]. However, it should be noted that previous research suggests that in order to substantially decrease illness absence and therefore produce economic benefits, one of the measures that can be taken is to increase classroom VRs above the State standard [23].

This fact has been highlighted by the COVID-19 pandemic. According to the World Health Organization, as of 7 July 2021, there had been 3,997,640 deaths and 184,572,371 confirmed cases of COVID-19 reported globally [24]. Transmission of SARS-CoV-2 occurs when uninfected people are exposed to infectious respiratory fluids after contact with infected people [25]. Factors contributing to increased transmission include: loud speech volume; intense physical activity; lack of well-fitting face masks; large numbers of people in the same space; decreased interpersonal distance; increased emission and exposure time and poor indoor VR [26]. Moreover, recent research has shown that transmission can be aggravated in confined and poorly ventilated spaces. Indeed, Nishiura et al. [27] state that COVID-19 transmission can be up to 18.7 times higher in confined spaces than in open air spaces. Park et al. [28] suggested that cross-ventilation is more efficient compared to single-sided ventilation, and recommend cross-ventilation to minimise the possibility of infection in high-density public buildings. According to Dai and Zhao [29], for a classroom with a volume of 348 m^3^ and for an exposure period of 2 h, to keep the probability of infection below 1%, a VR of two Air Changes per Hour (*ACH*) with masks and seven *ACH* without masks is necessary. 

Since students and teachers spend long periods each day in classrooms, these indoor spaces are risk environments for the airborne transmission of SARS-CoV-2 [30]. Consequently, measures adopted by governments to minimise the possibility of contagion included the closure of educational buildings. As a result, nearly half of the world’s students are still affected by this measure and more than 100 million additional children will fall below the minimum level of reading proficiency [31]. The United Nations Educational, Scientific and Cultural Organization (UNESCO) warns that it is crucial to prioritise education recovery in order to avoid a generational catastrophe [31]. Adopting effective mitigation strategies to control the risk of airborne infection and adapting educational-learning spaces are essential processes to mitigate the impact of educational building closures. The reopening of educational buildings has had many socio-economic implications in all countries, and therefore countries are taking actions to ensure that educational buildings are safe spaces. In this regard, the Spanish Government’s prevention guidelines require the use of well-fitted facemasks (a surgical mask is a minimum), reducing the volume of the voice in conversation, increased interpersonal distance and reduced contact time (e.g., reducing the occupation of indoor spaces) and improved ventilation in indoor spaces. Ventilation strategies are a key aspect of indoor spaces management in this context. In the case of natural ventilation, cross-ventilation (opening doors and/or windows on opposite sides) is recommended [26]. For mechanical ventilation, attention should be paid to the configuration of the system, to reduce the recirculation of air and increase/maximise outside air. The VR is measured by ACH. The recommended VR in indoor spaces for good air quality is 12.5 litres/second per person (L/s/p), which corresponds to approximately 5–6 ACH.

However, while these ventilation strategies ensure an optimal concentration of CO_2_ and other pollutants, they also have an impact on other important indoor variables in indoor environments. One of the most important in teaching‒learning spaces is the indoor acoustic environment, which is influenced by the natural and/or mechanical ventilation strategy selected [32]. In recent years, perceived acoustic quality in indoor environments has gained momentum and recent research has focused on indoor soundscapes [33,34] Acoustic design and strategies should include noise control and perceptual approach of the users in order to enhance people’s health and well-being [35,36]. In this sense, Tang [37] analysed available façade noise control strategies for introducing devices while improving natural ventilation in buildings. The findings of his study show that, in congested cities, protrusive devices such as balconies, lintels and fins are not effective noise screening devices for high-rise buildings (even with sound absorbers and/or reflectors). Active control installation and resonance-based devices often result in bulky systems, affecting the façade design and the effectiveness of natural ventilation strategies. Systems such as plenum windows and double-wall plenum structures are often useful as natural ventilation and noise control devices. In addition, research is being conducted on the development of new window devices. Fusaro et. al. [38,39] proposed a new metacage window which allows natural ventilation and noise reduction based on the principle of Snell’s Law. The used of this novel prototype showed an overall mean sound reduction of 15 dB within a bandwidth of 380 to 5000 Hz.

In this context, the management of natural ventilation strategies and their impact in the indoor acoustic environment is essential in the teaching‒learning spaces. Poor acoustic environments in classrooms affect learning achievements [40,41] as well as the academic, psychosocial and psychoeducational performance of students [42]. Moreover, these may cause voice problems [43] and physical stress in teachers [44], and have significant effects on word identification and intelligibility [45]. External noise sources to educational buildings as well as sources within the building (e.g., in facilities rooms, contiguous spaces, etc.) influence the background noise inside the teaching‒learning spaces. In order to achieve an adequate acoustic comfort and speech intelligibility to ensure the quality of educational processes the background noise level should not exceed the sound level of 35 dBA [46,47]. Therefore, acoustic comfort is critical in determining the quality of educational processes. This fact makes it necessary to evaluate the impact of the ventilation strategies on IEQ parameters such as IAQ and acoustic performance. This is the main general purpose of this research.

In this context, and given the 6 *ACH* values recommended in current Spanish public policies to prevent the transmission of COVID-19, the aim of this study was to characterise their impact on the variables conditioning IAQ and the indoor acoustic environment. The study assesses the need to define health protocols for ventilation in educational buildings that, in addition to identifying natural ventilation strategies with a VR value as close as possible to the required *ACH* value, take into account the background noise level. This will therefore ensure the quality of teaching and learning processes while maintaining the required ventilation protocols.

## 2. Methodology and Data Collection

**Figure 1:**
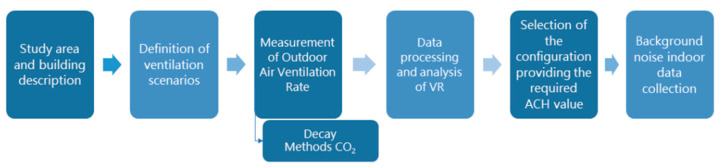
Diagram of the study’s methodological approach.

With the aim of characterising the impact of natural ventilation strategies on the variables conditioning IAQ and the indoor acoustic environment, natural ventilation efficiency was checked in three ventilation scenarios with different window and door opening configurations. Background equivalent continuous sound pPressure level (Leq) in dBA was also calculated from sound pressure levels measured in the configuration that provided sufficient VR through natural ventilation according to the current regulatory limit. This value was compared with the background equivalent continuous sound pressure level, measured in the closed doors and windows scenario. This section describes the study area, the data-collection methodology and the sensors used in the process. Figure 1 shows an overview of the study’s methodological approach.

### 2.1. Study Area and Building Description

**Figure 2:**
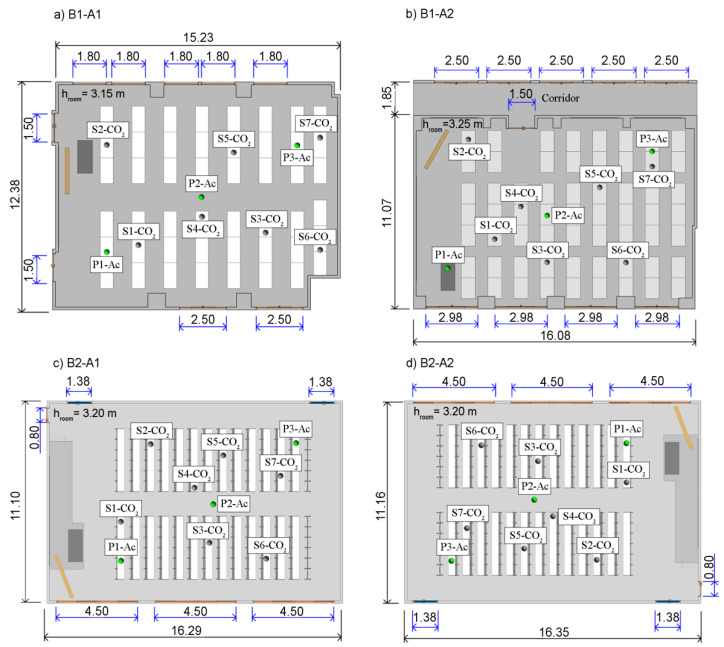
Location of the sensors during the experimental tests performed in each classroom; (**a**) B1-A1 classroom; (**b**) B1-A2 classroom; (**c**) B2-A1 classroom. (**d**) B2-A2 classroom; Blue dimensions indicate size of the openings; Black dimensions indicate sizes of the room; Green spheres indicate the position of the acoustic sensors; Grey spheres indicate the position of CO_2_ sensors (dimensions in meters).

The study comprises educational buildings from the Fuentenueva Campus of the University of Granada, located in Granada (Spain). The field measurements were conducted between March and April 2021 (spring season) in the Advanced Technical School for Building Engineering (built in 1972) and the Advanced Technical School for Civil Engineering (built in 2000) (Figure 2).

Face-to-face teaching was suspended at the University of Granada from October to January in response to COVID-19. The tests were carried out before the adaptation of the teaching spaces to the return to face-to-face teaching activities. For this purpose, ventilation and acoustic measurements were carried out in the newly adapted spaces. Granada is classified as a C3 zone by the Spanish Technical Building code CTE [48]. This zone is characterised by short, very hot and mostly clear summers and long, cold and partly cloudy winters. During the course of the year, the temperature generally varies from 0 °C to 34 °C and rarely drops below −4 °C or rises above 38 °C.

Two representative classrooms were selected for each building based on the data provided by the COVID-19 Action Plan developed by the University of Granada [49]. This plan defines institutional policies and guidance on occupational health and safety, which include: mandatory masks indoors, 50% occupancy, physical distancing (at least 1.5 m) and that indoor spaces must be ventilated naturally through open windows and doors. Within this framework, and in order to adapt the general measures established by the general action plan, the Academic Direction of each Technical School drew up an action plan adapted to their needs and to the characteristics of their spaces. The selection of these spaces took into account all the measures developed in this context.

Table 1 shows the characteristics of the classrooms. The process of characterisation and analysis starts with the selection of representative classrooms from the buildings of the campus. It should be noted that each selected classroom has a different orientation and that their geometry allows them to meet the requirements set out in the COVID-19 Action Plan. In addition, their different characteristics allow different ventilation strategies to be analysed: Classroom B1-A1 has windows on opposite sides, so natural cross-ventilation strategies can be assessed; Classroom B1-A2 is accessed through a corridor with windows, so cross-ventilation through corridors can be assessed; Classrooms B2–A1 and B2–A2 have identical geometries but are located on opposite sides of the building, such that ventilation strategies can be compared according to the location of the room.

### 2.2. Decay Method to Determine Air Change in Natural Ventilation in Classroom

The decay method can be used in unoccupied spaces using a tracer gas such as CO_2_. The aim of this method is to determine the *ACH*. In fact, the decay method consists of increasing the CO_2_ concentration by using a CO_2_ generation source (e.g., dry ice) [50] in the classroom until a homogeneous and well-mixed mixture is reached [16,51,52]. Subsequently (without source and unoccupied) the rate of decrease of the CO_2_ concentration under the different configurations is determined. The experimental test ends when the CO_2_ level approaches 37% of its original peak concentration above the background [51,52]. For this purpose, the CO_2_ concentration is measured at known times and the *ACH* can be estimated using Equation (1):

(1) $$ACH=\frac{-1*\;\ln\left(\frac{C_{end}-C_{outdoor}}{C_{start}-C_{outdoor}}\right)}{t_{end}-t_{start}}$$

where *C_end_* is the measured CO_2_ concentration at the end of the decay curve, *t_end_* is the end time of the decay curve, *C_start_* is the measured CO_2_ concentration at the start of the decay curve, *t_start_* is the end time of the decay curve and *C_outdoor_* is the measured CO_2_ concentration outside the building.

Otherwise, in order to fit a solution to the decay concentration process using a regression or other means, a sequence of CO_2_ concentrations over a portion of the decay period, $C_{t}$, is used as shown in Equation (2) [16]:

(2) $$C_{t}=\left(C_{Start}-C_{outdoor}\right)exp\left(-ACH*t\right)+C_{outdoor}$$

where t is the measurement time in hours. In addition, Equation (2) can be rearranged to be linear in time as (Equation (3)):

(3) $$ln\left(C_{t}-C_{outdoor}\right)=-ACH*t+ln\left(C_{Start}-C_{outdoor}\right)$$

where $C_{Start}$ is the steady-state CO_2_ concentration at the start of the test. The estimated *ACH* is the slope of the regression of *ln*($C_{t}$ − $C_{outdoor}$) against time *t*. 

In this study, this method was applied for the VR characterisation of three configurations for each classroom. The values obtained were used to compare the *ACH* provided by each configuration. In addition, and given that the re-opening guidelines [26] recommend a ventilation rate of 12.5 litres per second and person to achieve good air quality (corresponding to approximately 5–6 ACH), the configuration providing the required *ACH* value was selected.

### 2.3. Background Noise Indoor Data Collection

In order to characterise the indoor acoustic environment in different configurations of natural ventilation strategies, the sound pressure level of the background noise was measured in the different configurations. For this purpose, a two-phase methodology was followed: in the first phase, the background noise was measured in the classroom with all doors and windows closed. Subsequently, in phase two, the background noise was measured with the natural ventilation configuration selected based on the experimental results of the decay method previously obtained (i.e., the configuration that provided the required *ACH* value).

During the field measurement period, three acoustical measurements were made at three seat locations in the classroom (front, middle and back) in both phases, resulting in nine measurements in each phase. The locations were selected because they were typical listener positions inside the classroom. The measurements were recorded at least 1.2 m away from the ground, 0.7 m between measurement positions and at 0.5 m. away from any wall, ceiling or ground surface, in compliance with the UNE-ISO 1996-2:2020 [53] recommendations (details about the instrument and positions are shown in Section 2.4 and Figure 2). Each measurement consists of a binaural recordings signal, which contains background noise and has a duration above 15 min. This minimum measurement time interval was selected because previous studies have identified that activity background noise level measured for a long time (4 h) was not found to be statically different from the values obtained over 15 min [54,55]. The measurements were recorded at the ear position using a head-torso manikin (height: 1.30 m) located in the listener positions previously selected. The manikin was stably fixed to perform the recordings in a stationary condition in order to avoid additional noise. The manikin’s head was oriented towards the typical teacher’s position in the classroom.

The continuous equivalent sound pressure level (Leq) of each acoustical measurement was calculated as the averaged equivalent-energy of the sound pressure levels from the left and right channels during the measuring time. Based on these measurements, an energy averaging of the acoustic measurement in each configuration was performed with the aim of obtaining a sound-level value (dBA) representative of each configuration.

The obtained values were then compared with the limits for the ambient noise level for teaching‒learning spaces recommended by the World Human Organization (WHO) [46] and ANSI/ASA S12.60-2010/Part 1 [47]. Both organisations recommend sound-level values below 35 dBA.

### 2.4. Sensors and Data Collection

The HOBO^®^ MX1102 logger was used to measure the CO_2_ concentrations in the classroom. The instrument has a measurement range from 0 to 5000 ppm (accuracy ± 50 ppm ± 5% of reading at 25 °C, less than 90% RH non-condensing and 1.013 mbar). The sensing method is non-dispersive infrared (NDIR) absorption. Regarding the acoustical signals recordings, these were made using a Squadriga I recorder and BHS I headset/microphone unit. The sampling rate of the external microphones was 48 kHz. Maximum sound pressure level of 130 dB_SPL_ and frequency response of 4 Hz to 20 kHz.

Figure 2 shows the position of the sensors in the experimental tests for each classroom. Seven HOBO^®^ MX1102 sensors were used during the decay method experimental tests, numbered in Figure 2 as sensor S1-CO_2_ to sensor S7-CO_2_. With regard to the acoustic measurements, they were performed in the locations P1-Ac, P2-Ac and P3-Ac (front, middle and back position in the audience respectively).

One of the fundamental requirements established in the COVID-19 Action Plan elaborated by the University of Granada was to establish natural ventilation through open windows and doors, even in adverse weather conditions [49].

For this reason, different scenarios of window and door opening combinations were selected to generate each configuration. Three types of configurations were defined for each of the four selected classrooms (Table 2). Experimental tests were carried out in order to evaluate the configuration that provides sufficient ventilation according to the COVID-19 standards. In addition, the impact of the selected configuration on the acoustic comfort was evaluated.

## 3. Results

In the next sections, results are presented for the three configuration scenarios of the four classrooms previously described. Firstly, each section shows the data obtained from the experimental tests of the decay method and the average *ACH* results. Subsequently, the background noise sound pressure levels Leq obtained in two different ventilation scenarios are shown: (1) doors and windows closed; and (2) the natural ventilation configuration that provides the *ACH* value required (based on the decay method experimental results previously obtained).

### 3.1. Building 1—Classroom A-1 (B1-A1): Windows-Based Natural Cross-Ventilation Strategies—East Orientation

**Figure 3:**
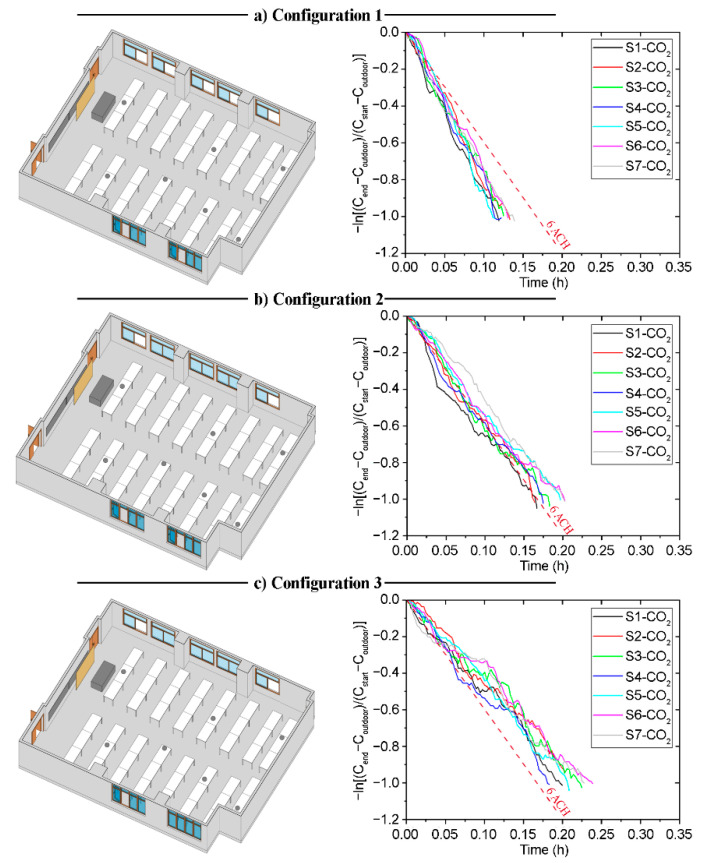
Configuration schemes and decay curves in Classroom B1-A1; (**a**) Configuration 1; (**b**) Configuration 2; (**c**) Configuration 3.

Figure 3 shows the decay methods results obtained for the three different configurations selected for the classroom B1-A1. In addition, the regression of ln(C1-CR) against time t is shown in Table A1 in Appendix A. Differences between the data recorded by each sensor are observed for the three tested scenarios. These are mainly due to the different relative positions of the sensors from the windows and doors, and may also derive from the indoor air currents. This fact is applicable also to all the tested natural ventilation scenarios shown in the following sections.

**Figure 4:**
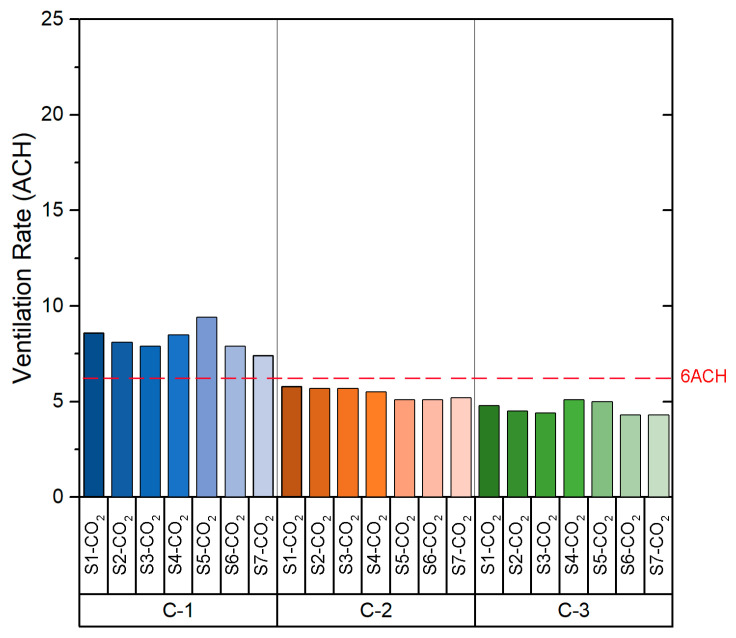
Ventilation rate (ACH) in Classroom B1-A1.

Based on the values shown in Table A1, the slope value obtained in the fitting curve of each case indicates the *ACH* value for the configuration measured at each point. As can be seen in Figure 4, which shows the *ACH* obtained in each configuration, the ACH values obtained are homogeneous. It should be noted that C-1 configuration is the one that provides the highest number of ACH. The *ACH* values in C-1 varied from 7.4 to 9.4 with a mean of 8.3 ± 0.6 per hour, whereas configuration C-3 shows the lowest ventilation rates, from 4.3 to 5.1 with a mean of 4.6 ± 0.3 per hour. Following the recommendations of the Spanish Ministry of Health [26], the recommended ventilation rate for indoor spaces (such as classrooms) is a minimum of 6 ACH. As we can see in Figure 4, the configuration that satisfies this premise is configuration C-1 (all windows opened and main door opened), in which the ventilation rate is higher than the 6 *ACH* value for all sensors.

**Figure 5:**
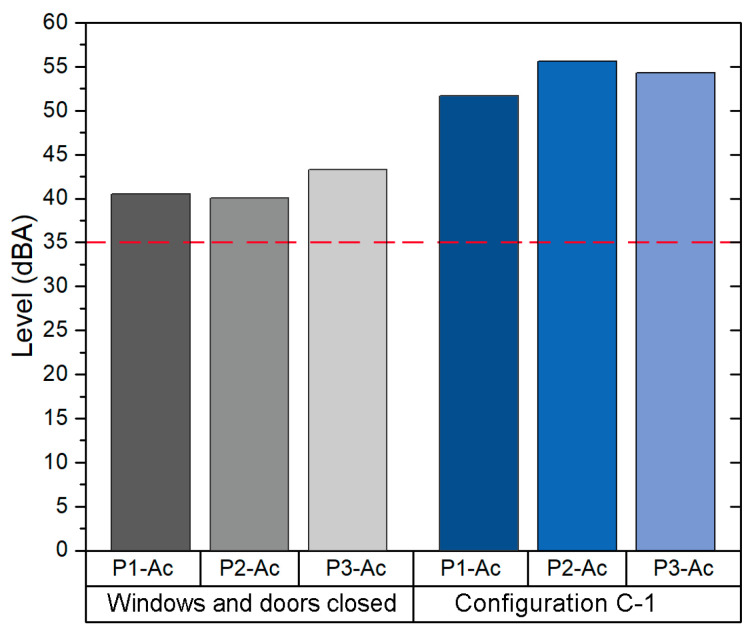
Background noise levels in classroom B1-A1.

Since configuration C-1 provides an *ACH* value above 6, it was selected in order to evaluate the background noise in this scenario. Hence, the background noise was measured in the following two configurations: (1) windows and door closed and (2) configuration C-1. As shown in Figure 5, the background Leq in the C-1 configuration is 12 dBA above the Leq measured in the same classroom with windows and door closed. 

The background noise Leq for the C-1 was 54.1 dBA. This value is above the background noise Leq with windows and door closed (41.5 dBA) and the value recommended by WHO (35dBA). Exposure to traffic noise is the main problem in this classroom, since it is located in the east façade of building 1, close to the main street of this district. The traffic noise has a high impact on the background noise of the classroom, since in order to achieve an adequate VR it is necessary to open all windows and the main door.

### 3.2. Building 1—Classroom A-2 (B1-A2): Cross-Ventilation through Corridors Strategies—West Orientation

**Figure 6:**
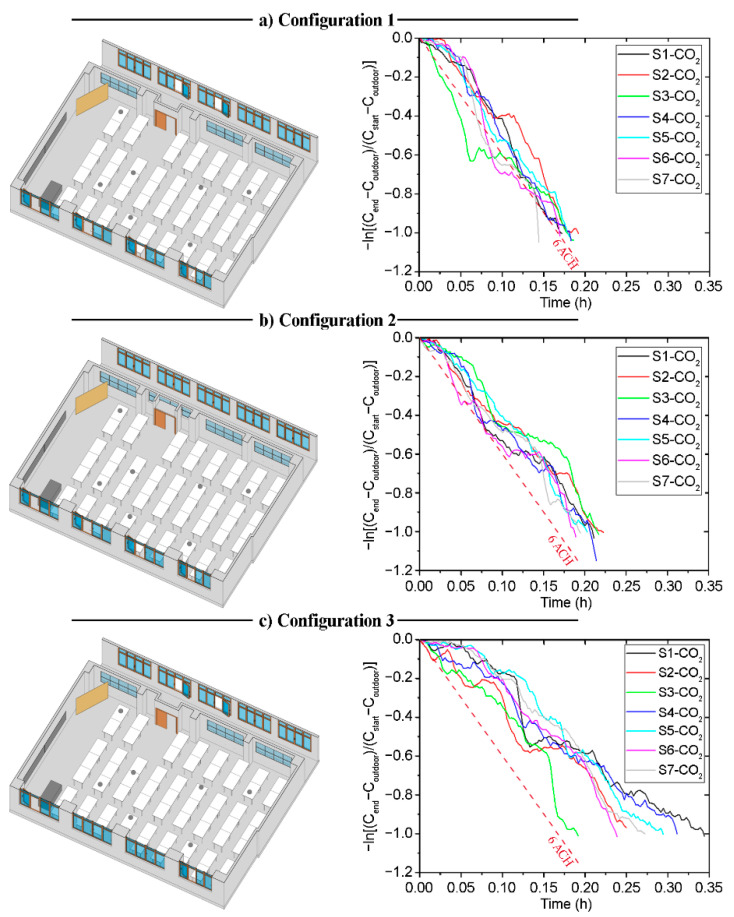
Configuration schemes and decay curves in Classroom B1-A2.; (**a**) Configuration 1; (**b**) Configuration 2; (**c**) Configuration 3.

The experimental results obtained in the tests performed in the classroom B1-A2 are shown in this section. This classroom is characterised by the fact that it can only generate natural ventilation through the windows located on its west side and the main door on its east side. In this respect, the different natural ventilation strategies have been analysed, taking into account scenarios with different opening configurations of these windows, the opening of the door and the possibility of opening the corridor windows. The decay methods results for the three configurations are shown in Figure 6. In addition, the data are analysed by fitting a curve using linear regression, as shown in Table A2 in Appendix A.

**Figure 7:**
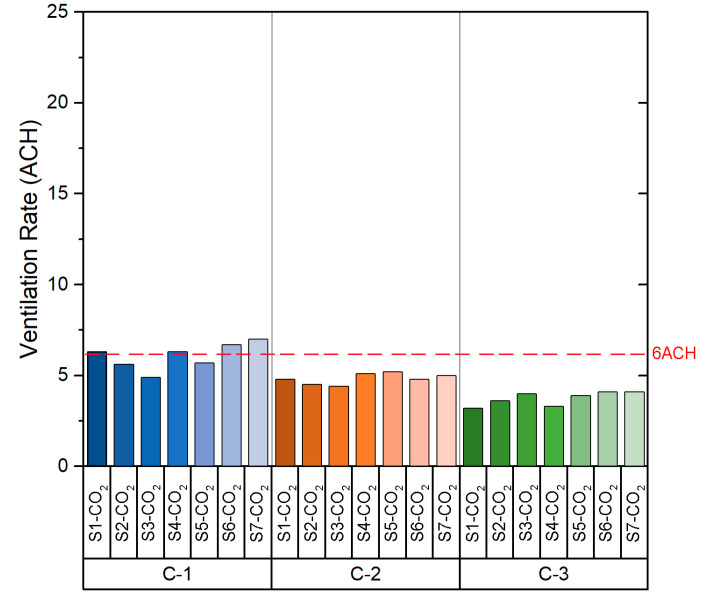
Ventilation rate (ACH) in Classroom B1-A2.

The *ACH* values obtained from each sensor are shown in Figure 7. As was seen in the case of the classroom B1-A1, the *ACH* values are homogeneous between the sensors in each configuration. If the data obtained for each configuration are compared, it is possible to appreciate that the *ACH* is higher in configuration C-1. The values obtained in both configuration C-2 and configuration C-3 are similar. However, configuration C-2 and C-3 provide *ACH* values below the recommended *ACH* limits stated in the legislation related to COVID-19.

**Figure 8:**
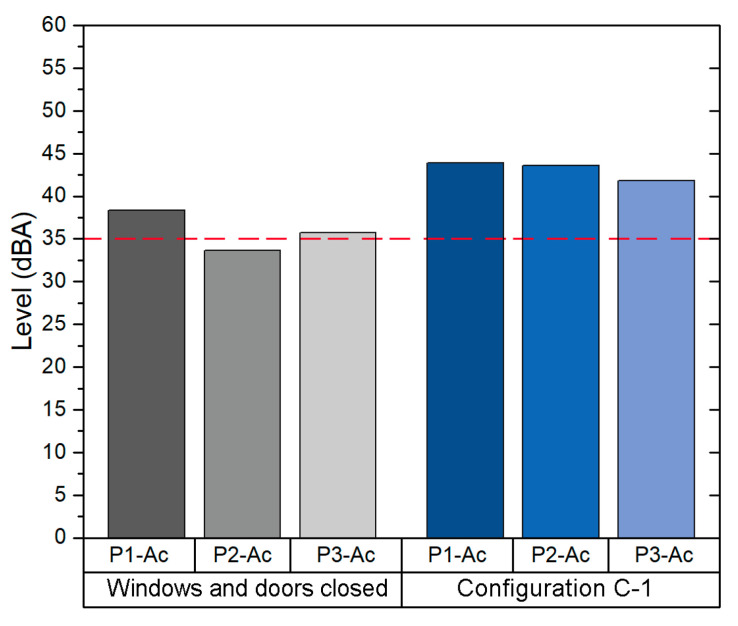
Background noise levels in classroom B1-A2.

In addition, the results obtained from the field acoustic measurement are shown in Figure 8. Since the C-1 configuration (i.e., all windows opened, main door opened and the corridor windows opened) provides an *ACH* higher than the recommendation set by the ministry guidelines (6 ACH), the background Leq was analysed first in the scenario with closed windows and door, and then in the scenario of configuration C-1. The average Leq value obtained in configuration C-1 was 44.5 dBA, 6.4 dBA above the Leq in the configuration of closed windows and door and also above the value recommended by the WHO (35 dBA).

In contrast to the classroom B1-A1, classroom B1-A2 is orientated towards a green zone in the opposite façade and a corridor. In this case, the dominant source of background noise is the noise generated by the students themselves when interacting with university activities.

### 3.3. Building 2—Classroom A1 (B2-A1): Natural Cross-Ventilation Strategies—North Orientation

**Figure 9:**
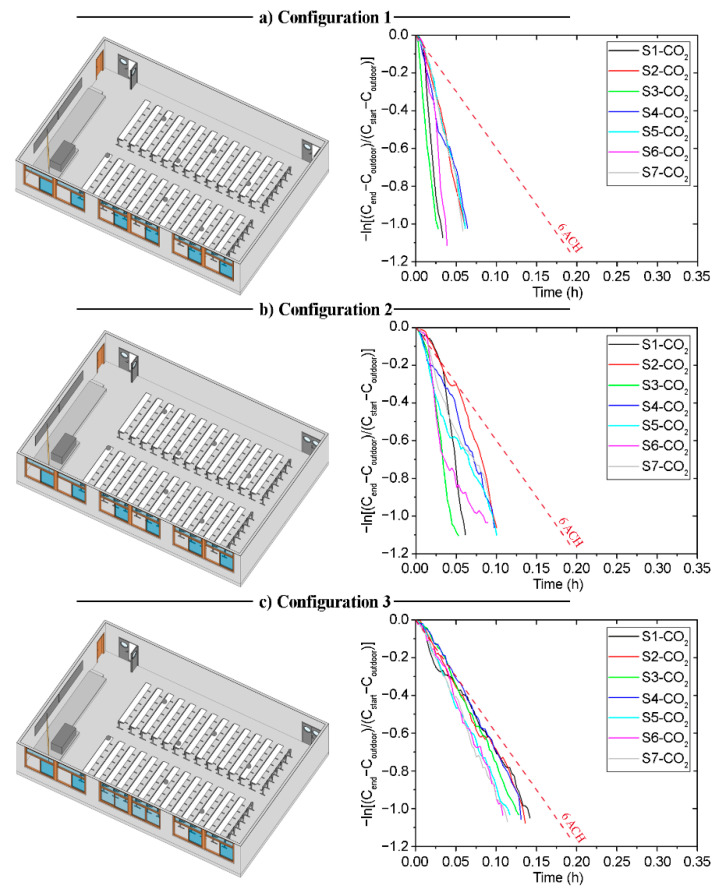
Configuration schemes and decay curves in Classroom B2-A1.; (**a**) Configuration 1; (**b**) Configuration 2; (**c**) Configuration 3.

Classroom B2-A1 is characterised by two doors located at the ends of one of its side walls. This wall is parallel to the side west wall, which contains the only windows in the room. These characteristics have been taken into account in the analysis of the different natural ventilation strategies. The field measurements were used to analyse the *ACH* in each configuration. The data obtained are shown in Figure 9 and Table A3 in Appendix A.

**Figure 10:**
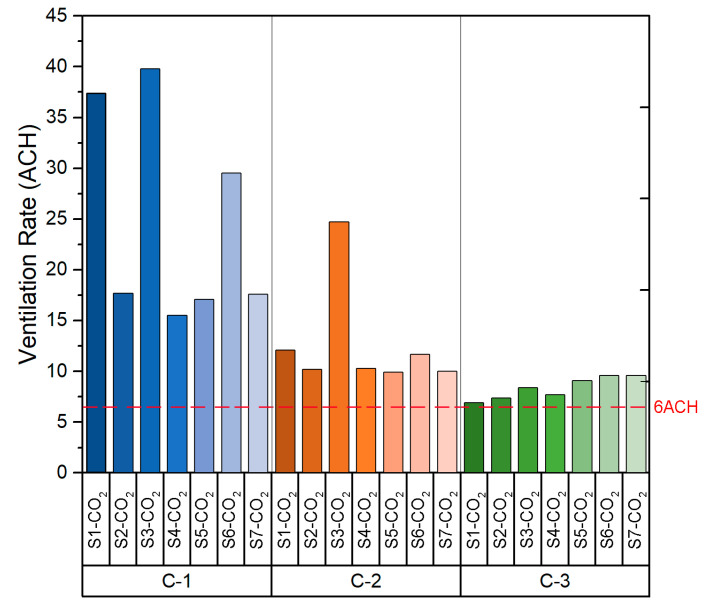
Ventilation rates (ACH) in Classroom B2-A1.

Figure 10 shows the *ACH* value obtained from the sensors for each of the configurations. Configuration C-1 provides *ACH* values far above the other configurations. The range of values obtained depends on the relative location of the sensors within the room. In this case, all configurations provide an *ACH* above the minimum set by the ministry’s guideline recommendations. However, the decay rate of the CO_2_ concentration in the case of configuration C-1 is caused by the air currents when opening the windows and doors. While such a high *ACH* is very safe, a high airflow through the windows may affect the comfort of the users. A configuration such as C-3 is preferable, which provides an *ACH* higher than the minimum set out in the guidelines, ensuring that ventilation will not impact on the performance and comfort of the students.

**Figure 11:**
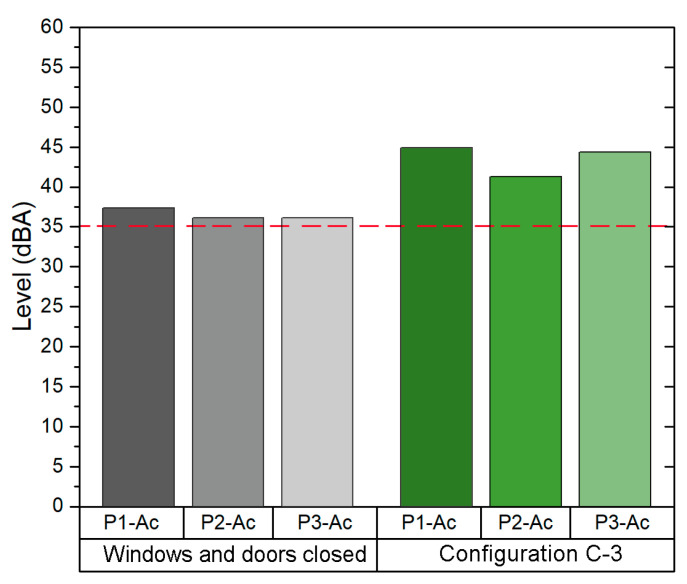
Background noise level in Classroom B2-A1.

In addition, the values obtained from the acoustic field measurements are shown in Figure 11. These values were measured in two scenarios: first closed windows and door, and then in the scenario with the configuration C-3 (i.e., only windows at the end opened and main door opened). The average background Leq value obtained in the measurement of the configuration C-3 is 43.7 dBA, 7.2 dBA higher than the value obtained in the measurement where the windows and doors were closed.

This classroom is orientated to other areas of the university campus and, as well as the B1-A2 classroom, the dominant source of background noise is the sound from university facilities. In this case, it is the only classroom studied in which it is not necessary to open all the windows to achieve the recommended VR (only windows at the end). This is reflected in the result obtained from monitoring the middle position, where the background noise is lower compared to the front and back positions.

### 3.4. Building 2—Classroom A2 (B2-A2): Natural Cross-Ventilation Strategies—South Orientation

**Figure 12:**
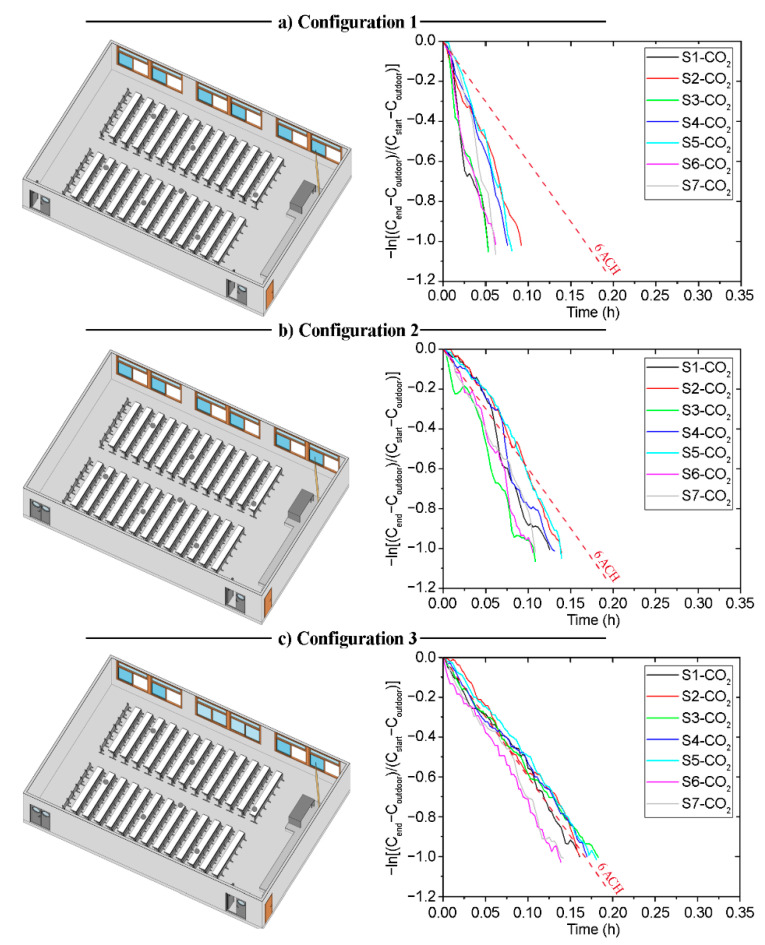
Configuration schemes and decay curves in Classroom B2-A2.; (**a**) Configuration 1; (**b**) Configuration 2; (**c**) Configuration 3.

The architectural characteristics of classroom B2-A2 are similar to those of classroom B2-A1. Both classrooms are located on the second floor of the ETSICCP building, but on opposite sides. Therefore, the only difference is the orientation of the room and its location in the building. In this case, classroom B2-A2 has the windows located on the west side, which is the opposite of the location of the windows in classroom B2-A1. The configurations selected for the natural ventilation strategy tests were the same as in classroom B2-A1 (see Table 2). The results obtained are shown in Figure 12 and Table A4 in Appendix A.

**Figure 13:**
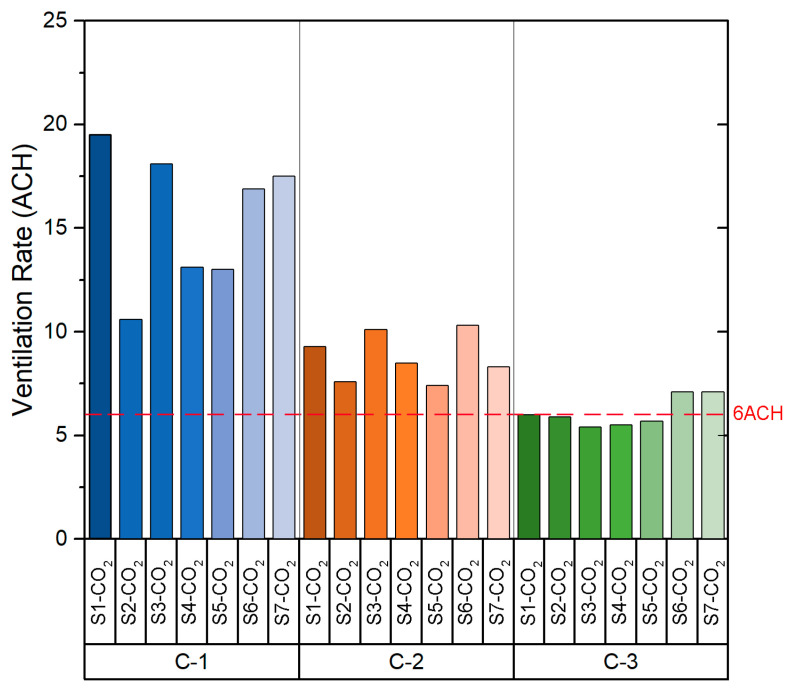
Ventilation rate (ACH) in Classroom B2-A2.

The *ACH* values obtained from the field measurements are shown in Figure 13. As in the case of class B2-A1, all three natural ventilation configurations provide *ACH* above the minimum set in the standard recommendation. However, the *ACH* values obtained in configuration C-1 are lower than those obtained in room B2-A1 with the same configuration. This is due to the orientation of the room, as the air currents are higher on the west façade of the building where room B2-A1 is located.

**Figure 14:**
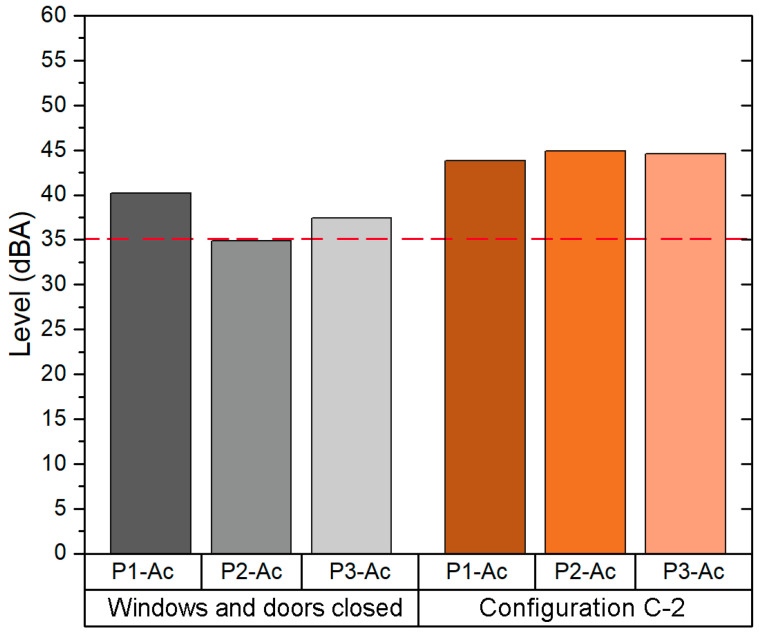
Background noise level in Classroom B2-A2.

Regarding the background noise sound pressure level values, these were measured first with the windows and doors closed, and secondly with configuration C-2 (i.e., all windows opened and the main door opened). The average Leq value obtained with configuration C-2 (44.5 dBA) is above that obtained with windows and doors closed (6.4 dBA). Figure 14 shows the results.

This classroom is similar in both geometry and predominant background noise to the classroom analysed in Section 3.3 (classroom B2-A1). In this sense, although both classrooms are in the same building, since their orientations are opposite the ventilation strategies to achieve the required VR are different for each of them. Thus, since in this classroom it is necessary to open all the windows (unlike B2-A1, where only windows at the end had to be opened), the background noise is slightly higher.

## 4. Discussion

The classrooms selected in this study are representative of the classrooms’ typology in Building 1 (Building Engineering School) and Building 2 (Civil Engineering School) of the University of Granada during return to teaching activity. For this purpose, field measurements were carried out to test three different configurations in the four selected classrooms.

The results obtained from the experimental tests in Building 1 showed that, in classroom B1-A1, the configuration that provides the lowest average *ACH* value is configuration C-3 (4.6) and the highest average value is configuration C-1 (8.3). In the case of the classroom B1-A2, the configurations providing the lowest and highest average value of *ACH* are configuration C-3 (3.7) and C-1 (6.1) respectively. With regard to the results obtained from the experimental analysis in Building 2, in the case of classroom B2-A1, configuration C-3 provides the lowest mean *ACH* value (8.4) and configuration C-1 provides the highest mean *ACH* value (24.9). In the case of classroom B2-A2, the configuration providing the lowest average *ACH* value is configuration C-3 (6.1) and the highest average *ACH* value is configuration C-1 (15.5).

As can be seen, the VR depends on the local and particular conditions of each indoor space. In this context, the configuration chosen among the three analysed in each classroom was the one that meets the minimum ventilation requirements. The configurations selected for classrooms B1-A1, B1-A2, B2-A1 and B2-A2 were configurations C-1 (all windows opened and main door opened), C-1 (all windows opened, main door opened and the corridor windows opened), C-3 (only windows at the end opened and main door opened) and C-2 (all windows opened and the main door opened) respectively. This decision is based on ensuring that the *ACH* value is sufficient to guarantee that the space is safe, although there may be variability in the *ACH* value due to possible variations in environmental conditions.

Once the natural ventilation configuration was selected for each classroom, an acoustic study was carried out to compare the normal classroom scenario (windows and door closed) with the chosen configuration of natural ventilation. As can be seen from the results obtained, since the background noise level should not exceed 35 dBA for good speech intelligibility, none of the classrooms met this acoustic quality recommendation. With regard to the comparison between the scenario of closed doors and windows and the natural ventilation configuration selected, it was identified that the natural ventilation configuration causes an increase of between 6.4 dBA and 12.6 dBA in the background noise level of the classrooms analysed. The background noise is an important factor that affects the acoustic clarity and quality of teaching and learning process [56].

Background noise is closely related to the signal-to-noise ratio (SNR). In this sense, a high level of background noise can cause a low or negative SNR. Therefore, a poor SNR causes, on the one hand, difficulties for students having to understand the message. On the other hand, it also causes a higher vocal effort among teachers, as the speaker’s speech level has to be higher than the background noise level.

In fact, background noise becomes a problem that has a major impact on the current situation. Since the classrooms used for the return to campus are larger, and to ensure physical distance between students the distribution of students occupies all rows of seats, many students are in positions far away from the teacher. As a result, the signal‒noise ratio is very low in the rear positions, causing significant effects on reducing word identification and intelligibility.

The location and orientation of the classroom also influences the impact of the natural ventilation configuration on classroom background noise. This is evident in the results obtained for classroom B1-A1, which is oriented towards a dense traffic area and the background noise level was 54.1 dBA. Therefore, more factors than room size and ventilation strategy should be taken into account when choosing the classroom. The location and orientation of the classroom should be considered in order to reduce the impact of background noise on the teaching‒learning process. Consequently, the practical implications of the findings show that ventilation strategies management in educational buildings should consider the following design and operation guidelines:

- The classroom selection must take into account both the health recommendations and the impact of background noise. Priority should be given to selecting those indoor spaces that: 1) meet the health requirements (minimum distances, VR, etc.) and 2) (due to their location and orientation) have a background noise level that does not interfere with the teaching-learning activities.
- In those cases where it is not possible to meet the criterion stated in the previous point, an adaptation intervention must be carried out (i.e., installation of passive, active, automation-based or hybrid noise control devices). Noise control solutions for natural ventilation openings must ensure the required VR while also ensuring the background noise does not interfere with the performance of students and teachers.

The limitations presented in the study stem from the effect of indoor and outdoor environmental conditions (the local and particular conditions of each indoor spaces as well as the wind speed and outdoor temperatures). Additionally, this study follows the protocols stated by the Spanish Government and University of Granada prevention guidelines. One of this protocols is the IAQ management of both buildings is to ventilate (for at least 1 h before and after each class) by opening all windows. This procedure achieves indoor temperature and relative humidity levels similar to those outside, so the effect of these factors should be taken into account if different conditions would apply.

## 5. Conclusions

The aim of this study was to analyse the natural ventilation strategies through the configuration of window and door openings, in accordance with the recommendations established in the COVID Action Plan of the University of Granada, which complies with the recommendations while maintaining the maximum degree of comfort for the user. To this end, the impact of these measures on the acoustic environment of the classroom was analysed, so that both students and teaching staff maintain safe levels of protection against the transmission of SARSCOV-2 without affecting their teaching‒learning activities.

The results obtained show that a correct choice of configuration can satisfy the VR needs while ensuring that the indoor space is safe for the occupants. The measurements were carried out in four different classrooms with an occupancy per area ranging from 2.20 m^2^/student to 4.77 m^2^/student. These spaces were selected according to the COVID-19 contingency plan set up at the beginning of the 2020/2021 academic year in each university centre. The natural ventilation configuration that met the required *ACH* was chosen to assess the impact on background noise inside the classroom. The main results obtained were:

- Natural cross-ventilation is an effective strategy to achieve the *ACH* levels required to ensure that the indoor spaces meet the guideline recommendations for a safe return to campus.
- There are differences in the specific natural ventilation strategy depending on the configuration of classrooms and building orientation. Thus, for the classrooms in building B1 the configuration of all windows opened and main door opened should be selected no matter the type of possible ventilation (natural ventilation through windows or cross-ventilation through corridors). On the other hand, in B2 the specific configuration depends on the classroom type, i.e., all windows opened and main door opened in the case of south-orientated classroom, or only windows at the end opened and main door opened in the case of the case of north-orientated classroom achieve better results due to the different orientation of the building. This fact highlights the needs of performing specific studies to select the best strategy to implement natural cross-ventilation.
- The average VR value provided by the selected configuration for each classroom was 8.3 ACH, 6.1 ACH, 8.4 *ACH* and 8.8 *ACH* for classrooms B1-A1, B1-A2, B2-A1 and B2-A2, respectively. Therefore, the average *ACH* value is above 6 *ACH* in all the selected natural ventilation configurations.
- The background noise level is strongly affected by the selected natural ventilation configuration. The background noise levels with the selected natural ventilation configuration were between 43.2 and 54.1 dBA. As can be seen, all classrooms exceed the recommended 35 dBA background noise level limit for background noise in teaching spaces. Consequently, the teaching activity management has to take into account not only the ACH, but also its impact on the indoor environmental conditions such as the acoustic environment. Since a high value of background noise level can interfere with the teaching and learning process and even interfere with the performance of students and teachers, educational building administrators need to consider this issue. In those cases where in order to achieve a natural ventilation strategy that provides the required VR, the background noise level exceeds 35 dbA, building managers should make intervening adaptations (i.e., installation of passive, active, automation-based or hybrid noise control devices).

Since this research proves that the best strategies to achieve a VR value that complies with the standard imply a significant impact in other indoor environmental variables such as indoor noise levels, some actions to improve the indoor acoustic behaviour of classrooms are recommended. For example, the need of electroacoustic support to increase speech intelligibility, improving the acoustic conditioning of classrooms, increasing noise insulation with other classrooms and other common areas, and reinforcing the compliance of outdoor noise levels achieving the acoustic quality criteria prescribed for sensitive acoustic areas such as the educational ones. Therefore, the management, organization and planning for indoor spaces of educational buildings must not only ensure occupants’ safety, but also not influence the performance of teaching activities. Action plans are required that allow buildings’ administrators to achieve adequate natural ventilation strategies and implement effective noise reduction measures in indoor spaces.

Finally, future studies should focus on the environmental conditions of natural ventilation with occupancy in the classrooms, in order to evaluate not only the objective variables of the IEQ factors, but also the subjective variables associated with the perception and comfort of occupants with regard to the window and door opening configurations established.

## Figures and Tables

**Table 1:** Characteristics of the classrooms.

Building	Id Class	Area [m^2^]	Volume [m^3^]	Orientation	Occupation Pre-Covid-19 [Seats]	Occupation Ratio [m^2^/Student]	Occupation Covid-19 [Seats]	Occupation Ratio [m^2^/Student]
Building 1 (ETSIE)	B1-A1	175	524	East	96	1.82	48	3.27
	B1-A2	167	500	West	61	2.73	35	4.77
Building 2 (ETSICCP)	B2-A1	172	518	North	156	1.10	78	2.20
	B2-A2	174	522	South	156	1.12	78	2.24

**Table 2:** Configurations for natural ventilation strategic tests.

Classroom	Configuration	Doors and Windows Opening Combinations
B1-A1	C-1	All windows opened and main door opened.
	C-2	End windows opened and main door opened.
	C-3	Only windows at the end in west façade opened, the centre windows in east façade opened (“Y” configuration) and the main door opened.
B1-A2	C-1	All windows opened, main door opened and the corridor windows opened.
	C-2	All windows opened, main door opened and the corridor windows closed.
	C-3	Only the windows at the end opened and main door opened, and the corridor windows opened.
B2-A1	C-1	All windows opened and two doors opened.
	C-2	All windows opened and main door opened.
	C-3	Only windows at the end opened and main door opened.
B2-A2	C-1	All windows opened and the two doors opened.
	C-2	All windows opened and the main door opened.
	C-3	Only windows at the end and the main door opened.

## Data Availability

Data are provided upon request to the corresponding author.

## Appendix A

This section contains data obtained from the experimental tests of decay method and the average *ACH* results.

**Table A1:** Decay curves in Classroom B1-A1.

Sensor	Configuration 1 (C-1)			Configuration 2 (C-2)			Configuration 3 (C-3)		
	Regression	R2	ACH	Regression	R2	ACH	Regression	R2	ACH
Sensor 1	y = −8.5562x − 0.0116	0.99	8.6	y = −5.8032x − 0.0643	0.97	5.8	y = −4.8164x − 0.013	0.99	4.8
Sensor 2	y = −8.1303x + 0.032	0.99	8.1	y = −5.6655x − 0.0146	0.99	5.7	y = −4.5337x + 0.0213	0.99	4.5
Sensor 3	y = −7.8861x + 0.0128	0.99	7.9	y = −5.6654x − 0.0067	0.98	5.7	y = −4.4275x + 0.0039	0.98	4.4
Sensor 4	y = −8.4811x + 0.0253	0.99	8.5	y = −5.4554x − 0.0361	0.98	5.5	y = −5.0707x − 0.0076	0.97	5.1
Sensor 5	y = −9.4362x + 0.0788	0.99	9.4	y = −5.1062x − 0.0007	0.99	5.1	y = −4.9633x + 0.0372	0.99	5.0
Sensor 6	y = −7.8993x + 0.0444	0.99	7.9	y = −5.0867x − 0.0127	0.99	5.1	y = −4.2515x + 0.0065	0.98	4.3
Sensor 7	y = −7.3562x − 0.0178	0.99	7.4	y = −5.2405x + 0.0546	0.99	5.2	y = −4.3029x − 0.0154	0.97	4.3

**Table A2:** Decay curves in Classroom B1-A2.

Sensor	Configuration 1 (C-1)			Configuration 2 (C-2)			Configuration 3 (C-3)		
	Regression	R^2^	ACH	Regression	R^2^	ACH	Regression	R^2^	ACH
Sensor 1	y = −6.337x + 0.1217	0.96	6.3	y = −4.7516x + 0.0131	0.97	4.8	y = −3.2131x + 0.0578	0.96	3.2
Sensor 2	y = −5.5545x + 0.1072	0.96	5.6	y = −4.4593x + 0.0234	0.98	4.5	y = −3.6485x + 0.0048	0.96	3.6
Sensor 3	y = −4.9443x + 0.1088	0.92	4.9	y = −4.3944x + 0.062	0.95	4.4	y = −3.997x + 0.0927	0.97	4.0
Sensor 4	y = −6.314x + 0.1222	0.98	6.3	y = −5.0606x + 0.0484	0.98	5.1	y = −3.2628x + 0.0311	0.99	3.3
Sensor 5	y = −5.7117x + 0.0777	0.99	5.7	y = −5.1964x + 0.0938	0.98	5.2	y = −3.8595x + 0.1546	0.96	3.9
Sensor 6	y = −6.6585x + 0.1135	0.93	6.7	y = −4.7563x − 0.0159	0.95	4.8	y = −4.1027x + 0.1336	0.95	4.1
Sensor 7	y = −6.9932x + 0.1164	0.94	7.0	y = −5.0269x + 0.0316	0.97	5.0	y = −4.0927x + 0.1599	0.96	4.1

**Table A3:** Decay curves in Classroom B2-A1.

Sensor	Configuration 1 (C-1)			Configuration 2 (C-2)			Configuration 3 (C-3)		
	Regression	R^2^	ACH	Regression	R^2^	ACH	Regression	R^2^	ACH
Sensor 1	y = −37.373x + 0.3503	0.97	37.4	y = −12.071x − 0.1136	0.95	12.1	y = −6.9297x − 0.0104	0.99	6.9
Sensor 2	y = −17.728x + 0.0923	0.99	17.7	y = −10.227x + 0.1121	0.96	10.2	y = −7.4124x + 0.0203	0.99	7.4
Sensor 3	y = −39.807x + 0.0103	0.99	39.8	y = −24.743x + 0.1323	0.97	24.7	y = −8.431x + 0.0709	0.99	8.4
Sensor 4	y = −15.465x + 0.008	0.98	15.5	y = −10.332x + 0.0293	0.99	10.3	y = −7.6596x + 0.0606	0.99	7.7
Sensor 5	y = −17.138x + 0.0901	0.99	17.1	y = −9.9033x − 0.0758	0.97	9.9	y = −9.0858x + 0.0245	0.99	9.1
Sensor 6	y = −29.454x + 0.1544	0.94	29.5	y = −11.694x − 0.1097	0.96	11.7	y = −9.5583x + 0.049	0.99	9.6
Sensor 7	y = −17.66x + 0.0902	0.98	17.6	y = −9.9647x − 0.0078	0.98	10.0	y = −9.58x + 0.0345	0.99	9.6

**Table A4:** Decay curves in Classroom B2-A2.

Sensor	Configuration 1 (C-1)			Configuration 2 (C-2)			Configuration 3 (C-3)		
	Regression	R^2^	ACH	Regression	R^2^	ACH	Regression	R^2^	ACH
Sensor 1	y = −19.547x − 0.0038	0.94	19.5	y = −9.2905x + 0.1220	0.97	9.3	y = −5.9718x + 0.0009	0.98	6.0
Sensor 2	y = −10.564x − 0.015	0.99	10.6	y = −7.6267x + 0.1165	0.97	7.6	y = −5.9458x + 0.0431	0.99	5.9
Sensor 3	y = −18.071x − 0.0384	0.96	18.1	y = −10.101x + 0.0051	0.97	10.1	y = −5.3937x − 0.0211	0.99	5.4
Sensor 4	y = −13.097x + 0.0481	0.99	13.1	y = −8.4722x + 0.1049	0.96	8.5	y = −5.5117x − 0.0074	0.99	5.5
Sensor 5	y = −12.97x + 0.0908	0.98	13.0	y = −7.3876x + 0.1053	0.97	7.4	y = −5.7052x + 0.0387	0.99	5.7
Sensor 6	y = −16.88x − 0.0424	0.97	16.9	y = −10.316x + 0.0732	0.98	10.3	y = −7.0921x − 0.0266	0.99	7.1
Sensor 7	y = −17.514x + 0.1204	0.97	17.5	y = −8.3386x + 0.0169	0.98	8.3	y = −7.0636x + 0.0025	0.98	7.1
